# Research on Space-Based Gravitational Wave Signal Denoising Based on Improved VMD with Parrot Algorithm

**DOI:** 10.3390/s25134065

**Published:** 2025-06-30

**Authors:** Jingyi Xi, Xiaolong Li, Yunqing Liu, Dongpo Xu, Qiuping Shen, Hanyang Liu

**Affiliations:** 1 Institute of Electronic Information Engineering, Changchun University of Science and Technology, Changchun 130022, China; 18844419310@163.com (J.X.); mzliuyunqing@163.com (Y.L.); xdp@cust.edu.cn (D.X.); sqp@163.com (Q.S.); lhy@163.com (H.L.); 2 Jilin Provincial Science and Technology Innovation Center of Intelligent Perception and Information Processing, Changchun 130022, China

**Keywords:** variational mode decomposition (VMD), Parrot algorithm (PO), gravitational waves, wavelet threshold (WT)

## Abstract

Gravitational wave (GW) signals are often affected by noise interference in the detection system; in order to attenuate the impact of detector noise and enhance the waveform characteristics of the signal, this paper proposes a space-based GW signal denoising method that combines the Parrot algorithm (PO) with the improved wavelet threshold (IWT) to optimize the variational mode decomposition (VMD). To address the challenge of selecting the number of modes K and the penalty factor α in VMD, PO is introduced to select the optimal parameters, achieving a good balance between global search and local optimization. The components after modal decomposition are divided into preserved modal components and noise modal components, and the IWT is introduced to further denoise the noise modal components; finally, the signal is reconstructed to achieve the purpose of denoising the GW signal. The algorithm is verified by the GW simulation signal and the measured signal. The experimental results show that the algorithm is superior to other algorithms in the noise separation of GW signals, significantly improves the SNR, improves the detection accuracy of GW, and provides a new technical means for the extraction and analysis of GW signals.

## 1. Introduction

The scientific prospects of space-based gravitational wave (GW) detection are far-reaching. GWs are space–time fluctuations caused by the accelerated motion or drastic changes of massive celestial bodies. The extraction and processing of GWs are of great significance to scientists’ exploration of the universe. However, the processing of space-based GWs faces huge challenges. The GW signal is weak and is easily disturbed by astrophysics, the detector itself, and the external environment [1,2,3]. In the process of GW detection, there are extremely high requirements for laser interferometer systems and complex signal-processing technologies [4]. Therefore, how to separate the GW signal from the signal containing strong noise and enhance the waveform characteristics of the GW signal is particularly critical.

The research on space-based GW detection and analysis is very extensive. Ya Zhao et al. studied and analyzed the coupling relationship between beam jitter caused by wavefront error and optical path noise and proposed a method to optimize the wavefront difference using the beam far-field transmission model, which effectively reduced the interference of noise on GW signals. However, this method requires precise measurement and correction of the wavefront error of the beam, and the precision and stability of the optical system are extremely high, which will greatly increase the complexity and cost of the system [5]. Collin Capano et al. first incorporated the spin effect into the search for GW signals and constructed a GW signal template library for binary black holes including spin effects, which improves the coverage of the template library and reduces the number of templates. However, it has higher requirements for computing resources and costs. In the high spin region, the inspection may fail to effectively detect the signal due to the mismatch between the template and the data, leaving the signal still hidden in the noise, resulting in deviations in sensitivity estimation [6]. Premkumar Duraisamy et al. used time series data to detect continuous GW signals by converting the signal from time domain to frequency domain based on a deep learning method of a convolutional neural network, which significantly improved the accuracy of signal recognition and maintained high detection performance under a low signal-to-noise ratio. However, the performance of convolutional neural network models is highly dependent on the quality and diversity of training data and is prone to overfitting during training [7].

In view of the difficulty in separating GW signals from noise and the optimization of variational mode decomposition (VMD) parameters during processing, this paper proposes a space-based GW signal denoising algorithm based on improved VMD with the Parrot algorithm. Firstly, according to the characteristics of GW signals, the Parrot algorithm (PO) [8] is introduced to adaptively adjust VMD parameters. Secondly, the GW signal is modally decomposed according to the parameters to obtain noise modal components and preserved modal components, in which the noise modal components are decomposed and denoised by improving the wavelet threshold. Finally, by reconstructing the processed signal components, the GW signal after noise reduction was obtained. Experiments show that the proposed algorithm realizes the adaptive adjustment of the denoising algorithm parameters, improves the signal-to-noise ratio of the signal and effectively reduces the root mean square error, improves the speed and accuracy of GW signal extraction, and shows excellent performance in signal denoising.

The European Space Agency (ESA) and the National Aeronautics and Space Administration (NASA) jointly proposed the Laser Interferometer Space Antenna Project (LISA). The space GW observatory uses three satellites 2.5 million kilometers apart to form an equilateral triangle that are connected by laser links [9,10]. Laser interferometry technology is the core technology for gravitational wave observations. The optical part of the laser interferometer uses two lasers to perform phase demodulation through an electro-optical modulator (EOM). After passing through an optical attenuator and a polarization beam splitter, the two laser beams interfere to generate a heterodyne beat signal, which is converted into four analog electrical signals after passing through a four-quadrant photodetector (QPD). After being processed and analyzed by a transimpedance amplifier (TIA), a variable gain controller (VGC), and an anti-aliasing filter (AAF), it is further processed by an analog-to-digital converter (ADC) and converted into a digital signal. The digital circuit part extracts phase information from the heterodyne beat signal through a digital phase-locked loop and demodulates data information from the modulated signal, so that the system can detect the tiny fluctuations caused by GWs by accurately measuring the phase difference between the light beams [11,12].

In the laser heterodyne interferometry system for GW detection, almost every transmission, amplification, and conversion process will generate noise, causing random distortion of the original signal [13]. In GW signal data analysis, especially GW data analysis, the removal of noise is one of the most challenging aspects. These noises are usually difficult to separate from the real signal [14,15], so a processing algorithm is needed to identify the noise and separate the GWs from the noise.

The GW signal is expressed as follows:

(1) f(t)=u(t)+n(t)

In the formula, f(t) represents the detector signal, u(t) represents the GW signal, and n(t) represents the noise signal.

The space GW detection system mainly includes three types of noise: front-end optical noise, analog circuit noise, and digital loop noise. Front-end optical noise, such as photon shot noise and light intensity coupling noise, has an obvious impact on the sensitivity and measurement accuracy of the detector. Shot noise is caused by the Poisson statistical characteristics of photons reaching the detector [16,17]. The fluctuation of the number of photons is manifested as shot noise, which is expressed as:

(2) $${\left(\delta L\right)}^{2}\approx \frac{hc\lambda B}{8\pi^{2}P}+\frac{8Ph\nu B}{m^{2}\omega^{4}c^{4}}$$

where L is the equivalent displacement noise, h is Planck constant (6.626 × 10^−34^ J∙s), c is the speed of light in vacuum, λ is the laser wavelength in vacuum, B is the observation bandwidth (1/2 t, where t is the observation time), P is the total optical power of the interference laser, m is the inertial mass of the test mass, and ω is the signal angular frequency variable.

Analog circuit noise, such as dark current noise and resistor thermal noise, is superimposed on the GW signal, which will cause aliasing and distortion of the GW signal. The power spectral density function of resistor thermal noise is expressed as:

(3) $$N(f)=\frac{\sqrt{4kT/R}}{I_{AC}}$$

where k is the Boltzmann constant, which is about 1.38 × 10^−23^ J/K; T is the absolute temperature of the resistor; R is the resistance of the feedback resistor; and $I_{AC}$ is the root mean square value of alternating current. Digital loop noise, such as quantization noise and crosstalk noise, will affect the frequency-locking accuracy of the GW signal. In the GW detection system, noise is a key factor affecting the detection accuracy and signal recognition ability [18,19,20]. Therefore, signal noise-reduction processing in the GW detection system is crucial.

## 2. Materials and Methods

### 2.1. Variational Mode Decomposition

GWs are affected by various background noises during transmission, and the amplitude is proportional to the inverse of the distance, which means that GWs gradually attenuate during transmission. The magnitude of the amplitude of GWs is typically on the nanometer or picometer scale [21]. It is particularly important to separate the signal from the noise through signal-processing technology to improve detection accuracy. VMD aims to decompose the signal into K modal component IMFs according to different frequencies. The K modal components are independent of each other in the frequency domain, and the modal component $u_{k}(t)$ is expressed as:

(4) $$u_{k}(t)=A_{k}(t)\cos[\phi_{k}(t)]$$

where $A_{k}(t)$ is the amplitude of each modal component, and $\phi_{k}(t)$ is the instantaneous frequency of each mode.

Variational problem construction. Each mode has a single center frequency and limited bandwidth. Under the constraint that the sum of each modal component is equal to the input signal, we find K modes so that the sum of the estimated bandwidths of each mode is minimized, so the constrained variational model is obtained:

(5) $$\left\{\begin{array}{l} \underset{\left\{u_{k}\right\},\left\{\omega_{k}\right\}}{\min}\left\{\sum_{k}{\left\|\partial_{t}[(\delta (t)+\frac{j}{\pi t})u_{k}(t)]e^{-j\omega_{k}t}\right\|}_{2}^{2}\right\}\\ s.t.\sum_{k}u_{k}(t)=f(t) \end{array}\right.$$

where $\left\{u_{k}(t)\right\}$ is the set of K modal component signals; $\omega_{k}$ is the set of center frequencies of K modal component signals.

Variational problem solution. In order to solve $\left\{u_{k}(t)\right\}$ and $\left\{\omega_{k}\right\}$ in the above formula, introducing Lagrange multiplier λ and quadratic penalty factor α, we convert the above constrained extremum problem into an unconstrained problem, and the augmented Lagrange expression is obtained:

(6) $$\begin{array}{l} L(\left\{u_{k}(t)\right\},\left\{\omega_{k}\right\},\lambda )=\alpha \sum_{k}{\left\|\partial_{t}[(\delta (t)+\frac{j}{\pi t})u_{k}(t)]e^{-j\omega_{k}t}\right\|}_{2}^{2}+\\ {\left\|f(t)-\sum_{k}u_{k}(t)\right\|}_{2}^{2}+\langle \lambda (t),f(t)-\sum_{k}u_{k}(t)\rangle \end{array}$$

The minimization problem is solved by alternating directions of multiplication operators, and $\left\{u_{k}(t)\right\},\left\{\omega_{k}\right\},\lambda$ is optimized iteratively. The expression of iterative update is:

(7) $${\hat{u}}_{k}^{n+1}(\omega )=\frac{\hat{f}(\omega )-\sum_{i\neq k}{\hat{u}}_{i}(\omega )+\hat{\lambda }(\omega )/2}{1+2\alpha {\left(\omega -\omega_{k}\right)}^{2}}$$

(8) $$\omega_{k}^{n+1}=\frac{\int_{0}^{\infty }\omega {\left|{\hat{u}}_{k}(\omega )\right|}^{2}d\omega }{\int_{0}^{\infty }{\left|{\hat{u}}_{k}(\omega )\right|}^{2}d\omega }$$

(9) $${\hat{\lambda }}^{n+1}(\omega )={\hat{\lambda }}^{n}(\omega )+\tau [\hat{f}(\omega )-\sum_{k}{\hat{u}}_{k}^{n+1}(\omega )]$$

From the decomposition steps of VMD, we know that the decomposition of the signal requires the selection of appropriate mode number K and penalty factor α. Too large a K will result in over-decomposition, while too small a K will result in under-decomposition. A α value that is too large will cause the loss of frequency-band information, while a value that is too small will lead to information redundancy. At present, the selection of these two parameters mostly depends on experience or trial and error. Therefore, the optimal K value and α value are selected according to the characteristics of the GW waveform.

### 2.2. Optimal Parameter Selection

The selection of VMD parameters is of vital importance [22]. PO can enhance the global search capability, avoid the local optimum problem, and help find the optimal parameters in the VMD processing. The PO is introduced to automatically optimize the two parameters of VMD through an intelligent search strategy. The unique characteristics of the PO in dealing with optimization problems are demonstrated by qualitative analysis and comprehensive experiments. By optimizing the K value and the value through the PO, the inefficiency of manual trial and error is avoided, and thus improve the overall computational efficiency.

By observing the key behaviors of Prrhura Molinae parrots, the PO can be divided into four processes: foraging, staying, communication, and afraid of strangers. The initialization formula of PO is as follows:

(10) $$X_{i}^{0}=lb+\mathrm{rand}(0,1)\cdot (ub-lb)$$

where rand(0, 1) represents a random number in the range, $X_{i}^{0}$ means the initial position of the i-th parrot, and ub and lb represent the upper and lower bounds of the search space constraints.

During foraging behavior, its movement follows the following expression:

(11) $$X_{i}^{t+1}=(X_{i}^{t}-X_{best})\cdot Levy(\dim)+\mathrm{rand}(0,1)\cdot (1-\frac{t}{Max_{iter}}{)}^{\frac{2t}{Max_{iter}}}\cdot X_{mean}^{t}$$

where $X_{i}^{t}$ is the current position, and $X_{i}^{t+1}$ is the subsequent update position. $X_{mean}^{t}$ represents the average position within the current population, and Levy distribution [23] is used to describe the parrot’s flight process. $X_{best}$ represents the best position searched from initialization to the present. $X_{mean}^{t}$ represents the best position from initialization to the current search, which is also the owner’s position; t represents the current number of iterations; and $M{\mathrm{ax}}_{iter}$ is the maximum number of iterations. The first part of the formula represents the movement based on the relative position of the owner, and the second part of the formula represents the observation of the position of the entire population to further determine the direction of the food.

The following formula represents the Levy distribution; to effectively balance the relationship between exploration and exploitation and improve the search efficiency and performance of the algorithm, γ is assigned a value of 1.5:

(12) $$\left\{\begin{array}{l} Levy(\dim)=\frac{\mu \cdot \sigma }{{\left|v\right|}^{\frac{1}{\gamma }}}\\ \mu ∼N(0,\dim)\\ \sigma ∼N(0,\dim)\\ \sigma ={\left(\frac{\Gamma (1+\gamma )\cdot \sin(\frac{\pi \gamma }{2})}{\Gamma (\frac{1+\gamma }{2})\cdot \gamma \cdot 2^{\frac{1+\gamma }{2}}}\right)}^{\gamma +1} \end{array}\right.$$

The following formula represents the process of staying behavior:

(13) $$X_{i}^{t+1}=X_{i}^{t}+X_{best}\cdot Levy(\dim)+\mathrm{rand}(0,1)\cdot \mathrm{ones}(1,\dim)$$

where ones(1,dim) represents the all-one vector of dimension dim, the second part of the formula represents the process of flying to the host, and the third part represents the process of randomly stopping at a certain part of the host’s body.

The communication behavior is represented as follows. The first part shows the communication behavior of individuals joining the parrot group, and the second part shows the behavior of individuals flying out of the group after communication:

(14) $$X_{i}^{t+1}=\left\{\begin{array}{l} 0.2rand(0,1)\cdot (1-\frac{t}{Max_{\mathrm{iter}}})\cdot (X_{i}^{t}-X_{mean}^{t}),P\leq 0.5\\ 0.2rand(0,1)\cdot \exp(-\frac{t}{rand(0,1)\cdot Max_{iter}}),P>0.5 \end{array}\right.$$

The fear behavior toward strangers is expressed as follows, where the second part represents the process of redirecting the flight toward the owner, and the third part represents the process of moving away from the stranger:

(15) $$\begin{array}{l} X_{i}^{t+1}=X_{i}^{t}+\mathrm{rand}(0,1)\cdot \cos(0.5\pi \cdot \frac{t}{Max_{\mathrm{iter}}})\cdot (X_{best}-X_{i}^{t})\\ -\cos(rand(0,1)\cdot \pi )\cdot {\left(\frac{t}{Max_{\mathrm{iter}}}\right)}^{\frac{2}{Max_{\mathrm{iter}}}}\cdot (X_{i}^{t}-X_{best}) \end{array}$$

The PO uses minimum envelope entropy as the fitness function, evaluates parameter performance, dynamically optimizes the K value, avoids modal aliasing or under-decomposition of VMD, and adaptively adjusts the α to balance the modal bandwidth constraint and convergence speed.

### 2.3. Processing of Noise Modal Components

After the above optimal parameter VMD decomposition, two modal components are obtained, one is the preserved modal components, and the other is noise modal components. In order to further process the noise modal components, the improved wavelet thresholding algorithm (IWT) is used. Since the hard wavelet threshold may cause a ringing effect or Gibbs phenomenon when reconstructing the signal, and the soft wavelet threshold may cause the loss of high-frequency details of the signal and cause edge blurring, the IWT is used to find a balance between the soft and hard thresholds. The expression is as follows:

(16) $$\hat{\omega }=\left\{\begin{array}{ll} \mathrm{sgn}(\omega )\cdot \sqrt{\left({\left|\omega \right|}^{2}-\lambda^{2}\right)} & \left|\omega \right|\geq \lambda \\ 0 & \left|\omega \right|<\lambda \end{array}\right.$$

IWT makes up for the discontinuity of the hard threshold function at ±λ and the shortcomings of the soft threshold function with constant error.

### 2.4. Overall Algorithm Process

First, the GW signal obtained by the detector is used as the input signal to initialize the parameters of VMD and PO and generate the initial parrot population. Secondly, according to the characteristics of the GW signal, the fitness function updates the parrot population and obtains the optimal parameters of VMD: the number of modes K and the penalty factor α. Next, the GW signal is subjected to VMD according to the optimized parameters to obtain the preserved modal components and the noise modal components. Then, the noise modal components are further denoised by IWT. Finally, the preserved modal components $u_{l}$ and the processed noise modal components $u_{k-l}$ are reconstructed to obtain the final denoised GW signal. The processing flow is as Algorithm 1:
**Algorithm 1.** Flowchart of space GW signals denoising based on PO-VMD-IWT algorithm.**1** **Input**: Original signal with noise *f***2** **Initialize**: *K*, α, {*u_k_*}, {*w_k_*}, λ, $X_{i}^{0}$, *t* = 1**3** PO algorithm initializes the configuration**4** **while** $p_{t}=\frac{a(t)}{\sum_{t=1}^{K}a(t)}$, $E_{p}=-\sum_{t=1}^{K}p_{t}\mathrm{lg}p_{t}$ minimal value**5** update ${\hat{u}}_{k}^{n+1}$, $\omega_{k}^{n+1}$, ${\hat{\lambda }}_{k}^{n+1}$, by using Equations (7)–(9)**6** update *K*, α, $X_{i}^{t+1}$, by using Equations (11) and (13)–(15)**7** t←t + 1**8** **if** *SampEn*, satisfy the threshold conditions of the preserved modes**9** extracting the signal *u_l_***10** **Elif** *SampEn*, satisfy the threshold conditions of the noisy modes**11** extracting the signal *u_k−l_***12** employing IWT filtering the *u_k−l_* by using Equation (16)**13** reconstruct *u_k−l_* and *u_l_***14** **end if****15** **Output**: final denoising gravitational wave signal *ǔ*

## 3. Experimental Analysis

The data used in this experiment are from the official website of the LIGO. The LIGO Observatory consists of two detectors, one in Hanford (H1), Washington and one in Livingston (L1), Louisiana, 3000 km away. When a GW passes through, it causes a slight distortion of space–time, resulting in a slight change in the length of the two arms of the interferometer. The two detectors work together to jointly analyze the data of the H1 and L1 detectors. When the waveform shapes detected by the two detectors are highly consistent, it can be proved that a GW signal has been detected. On 14 September 2015, the two detectors detected a “chirp” signal almost simultaneously. After rigorous noise elimination and theoretical analysis, it was confirmed that this was a GW signal generated by the merger of two black holes [24], marking the first time that humans have detected a GW signal.

The signals obtained by the detector are often mixed with a variety of complex noises. When transmitted and processed by devices such as the Quadrant Photo Detector (QPD), Trans-Impedance Amplifier (TIA), and Analog-to-Digital Converter (ADC), some noise is filtered out in the process, but residual noise components are still inevitable in the signal. Therefore, it is very important to conduct in-depth analysis of GW signals and effectively reduce noise, so as to reveal the physical properties of GW sources, enhance the identifiability of GW signals in background noise, and improve the sensitivity and reliability of detectors.

### 3.1. Analog Signal Experiment

**Figure 1:**
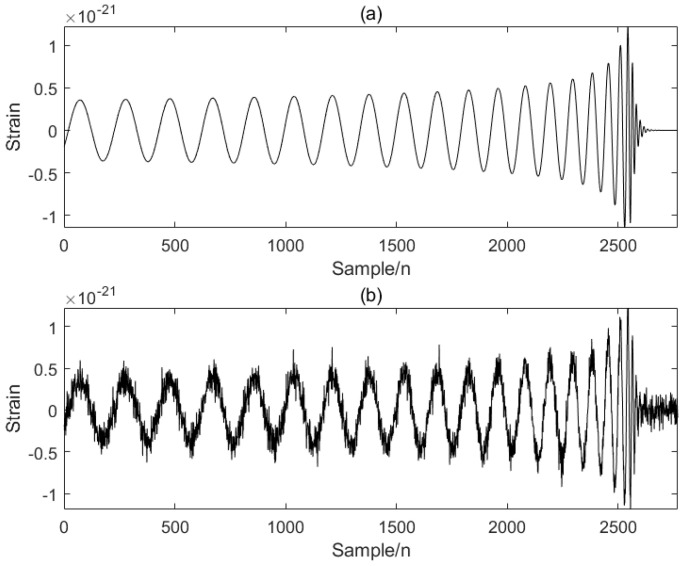
(**a**) Analog signal, (**b**) noise-added analog signal.

This simulation experiment uses simulated data of GW signals with a time length of about 0.6 s. In order to verify the effectiveness of the proposed algorithm, Gaussian white noise is added to the simulated signal. Gaussian noise is a common type of noise in GW detection [25], and its probability distribution is Gaussian distribution. Figure 1 takes the addition of Gaussian white noise as an example to simulate random interference such as thermal noise and electronic noise inside the detector.

**Figure 2:**
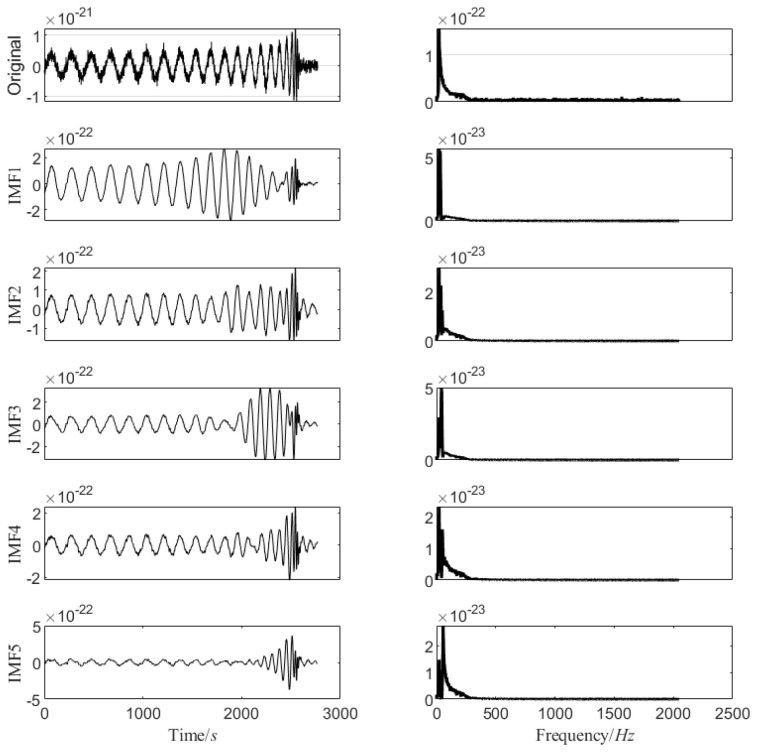
VMD modes and spectrogram of analog signal.

**Figure 3:**
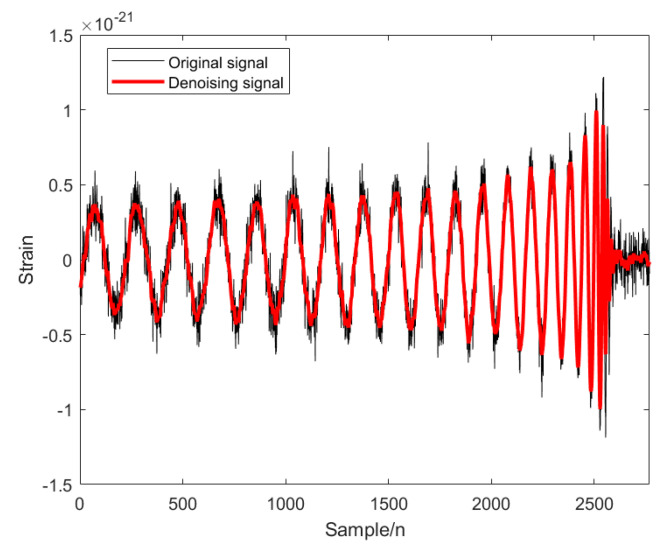
Analog signal after PO-VMD-IWT denoising: black is the original signal, and red is the denoising signal.

The modal decomposition number of VMD calculated by PO is 5, as shown in Figure 2. The simulated signal is decomposed into five modal components, and the sample entropy of each mode is calculated. The appropriate threshold is selected to divide the mode into the preserved modal components and the noise modal components. The modal component is smaller than the sample entropy threshold, and the modal component is larger than the sample entropy threshold. The IWT denoising algorithm is introduced to further denoise the noise modal components. Finally, the preserved modal components and the component denoised by the IWT are reconstructed to obtain the final denoised simulated GW signal, as shown in Figure 3 below.

**Figure 4:**
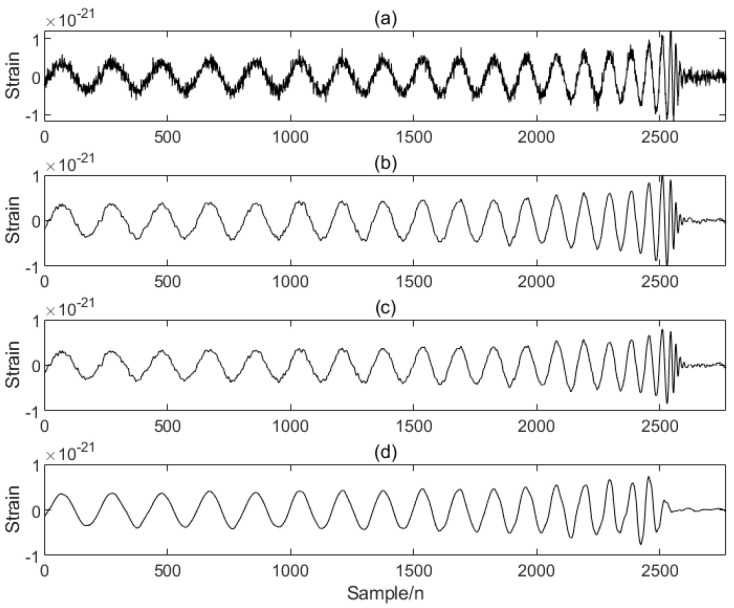
Comparison of PO-VMD-IWT and other algorithms: (**a**) original signal, (**b**) PO-VMD-IWT, (**c**) PO-VMD, (**d**) IWT.

In order to verify the excellence of the proposed algorithm, the processing results of the proposed algorithm are compared with those of other algorithms. As can be seen from the comparison Figure 4, the noise is significantly reduced after PO-VMD-IWT processing in this experimental method, and the waveform characteristics of the signal are retained while removing most of the noise. The processed data is smoother, and the data characteristics are most obvious; the noise is reduced after PO-VMD processing, but compared with the proposed algorithm, the noise removal effect is slightly inferior, and the smoothness and clarity are slightly lower; the IWT algorithm performs the worst in noise removal and signal retention, and the signal is obviously distorted.

**Figure 5:**
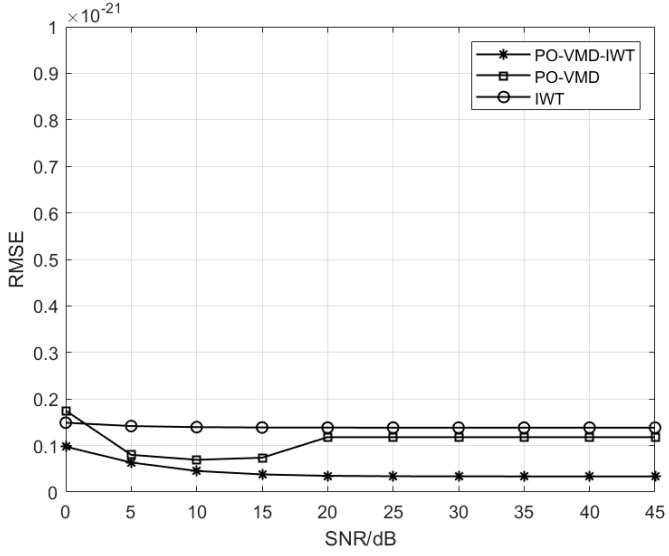
RMSE comparison of PO-VMD-IWT, PO-VMD, and IWT algorithm.

In order to check the proposed algorithm, different signal-to-noise ratios are given, and the root mean square errors of three different algorithms are calculated. As can be seen from Figure 5, the root mean square error of the proposed algorithm is smaller than that of the other two algorithms, and the root mean square error of the proposed algorithm is one order of magnitude different from that of IWT. The difference between the proposed algorithm and the reference signal is the smallest, which can verify the effectiveness, robustness, and accuracy of the proposed algorithm.

### 3.2. Measured Data Experiment

**Figure 6:**
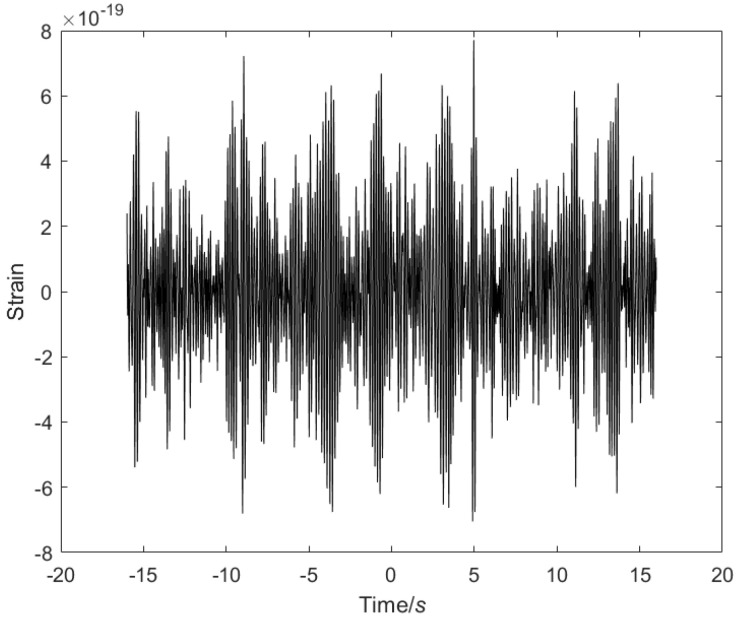
GW150914 detector signal.

GW150914 is the first GW event directly detected by humans. It originated from the merger of two black holes and has extremely important scientific significance. This experiment uses the GW150914 experimental data for analysis and research, as shown in Figure 6. According to the data characteristics, the most appropriate modal decomposition parameters are adaptively selected through the PO. Then, the GW signal is modally decomposed using the optimal parameters, the modal decomposition number K and the penalty factor. The preserved modal components and noise modal components are selected according to the sample entropy function threshold. Finally, the noise modal components are further denoised by the IWT, and the processed signal is recombined with the preserved modal components to obtain the final denoised GW signal.

**Figure 7:**
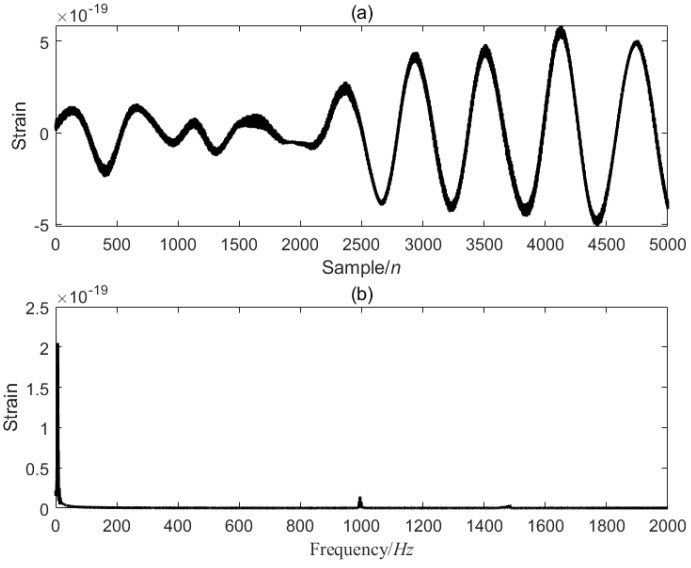
GW150914 signal [22001–27000] data segment. (**a**) is the time-domain signal, and (**b**) is the frequency-domain signal.

**Figure 8:**
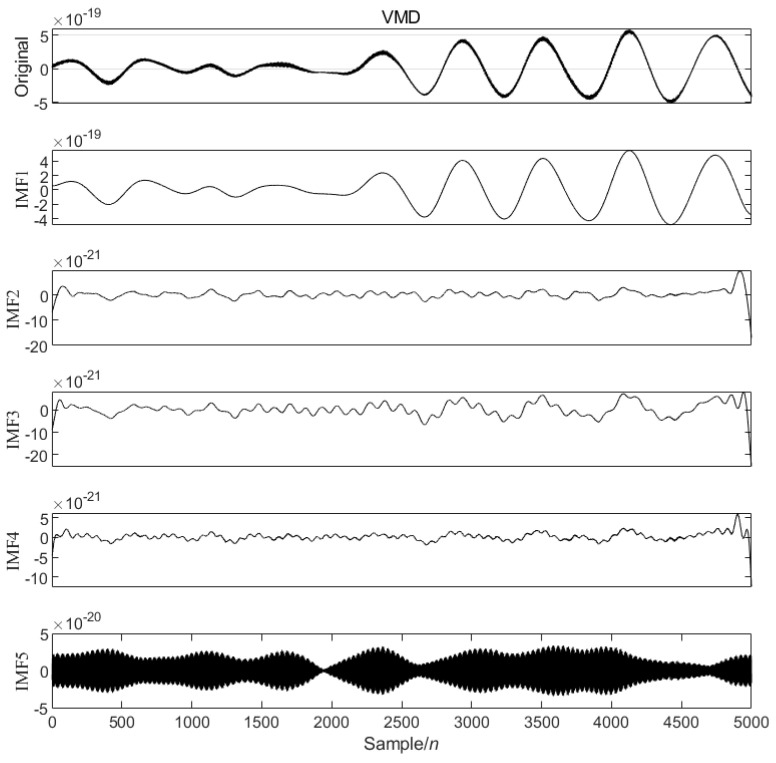
VMD modes of the data segment GW150914 [22001–27000].

Figure 7 is the first data segment [22001–27000] of the GW150914 signal, and the optimized VMD of the [22001–27000] data segment is performed, five modal components are decomposed, and the value of α is 1266.0714, as shown in Figure 8.

**Figure 9:**
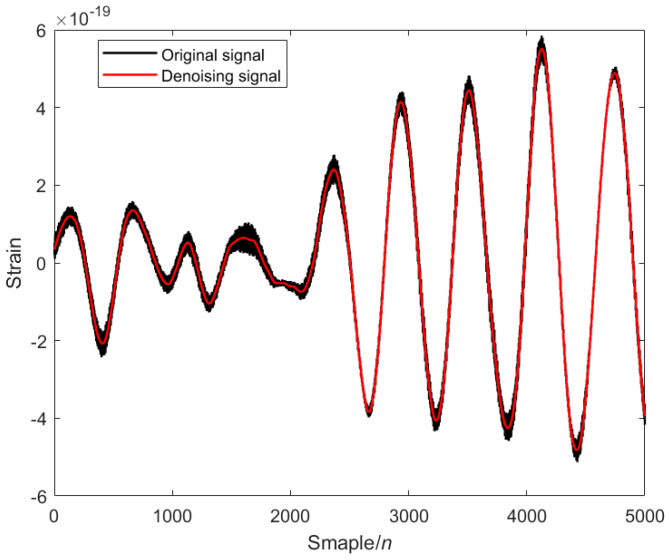
GW150914 signal [22001–27000] data segment noise-reduction result: black is the original signal, and red is the denoising signal.

From the comparison results of the original signal and the one after noise-reduction processing, in Figure 9 it can be seen that the quality of the [22001–27000] data segment has been significantly improved, most of the noise of the original GW signal has been effectively removed, and the waveform characteristics of the GW signal have been successfully retained.

**Figure 10:**
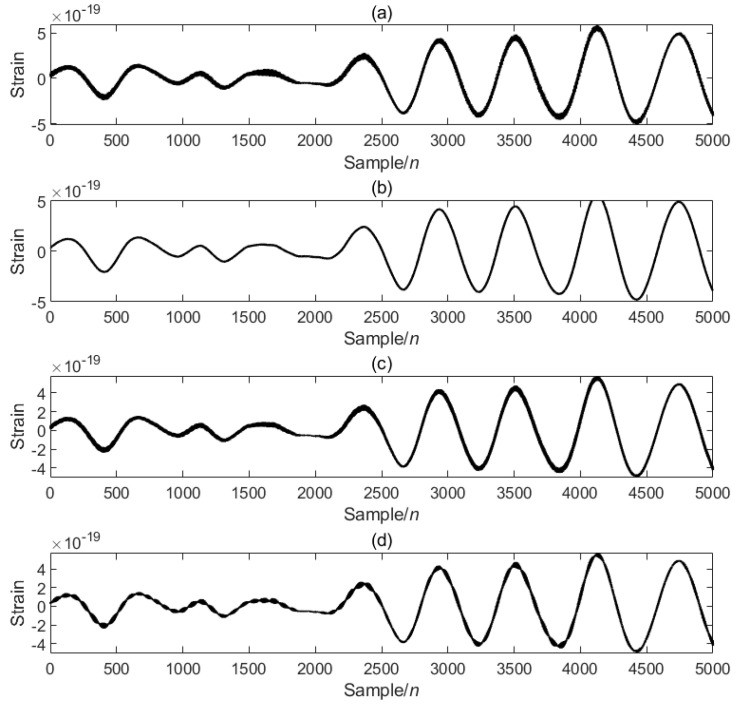
Comparison of various methods for the data segment GW150914 [22001–27000]: (**a**) original signal, (**b**) PO-VMD-IWT, (**c**) PO-VMD, (**d**) IWT.

From the contrast (Figure 10) of different algorithms, it can be seen that although the signal processed by PO-VMD can effectively suppress GW noise, the figure shows that there is still residual noise in some parts, especially in the high-frequency part of the signal; compared with other algorithms, IWT performs the weakest in noise suppression. It can be seen from the figure that the noise is still relatively obvious in the signal. In some places, the signal is over-smoothed, causing the loss of detailed information of the GW signal; the PO-VMD-IWT algorithm performs best in recovering GW signals. The noise in the signal is significantly reduced. While reducing noise, the main features and details of the signal are better retained, the waveform is clear, and the signal is smoother.

**Figure 11:**
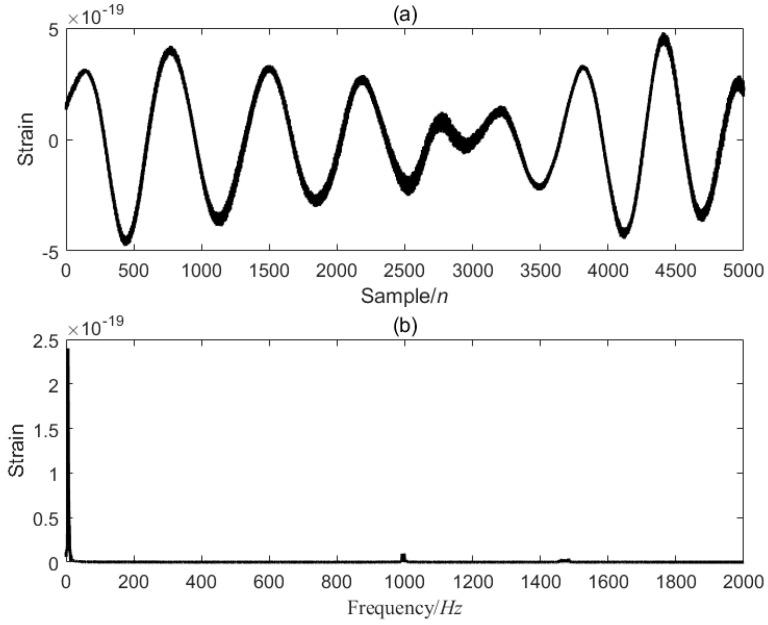
GW150914 signal [41001–46000] data segment. (**a**) is the time-domain signal, and (**b**) is the frequency-domain signal.

**Figure 12:**
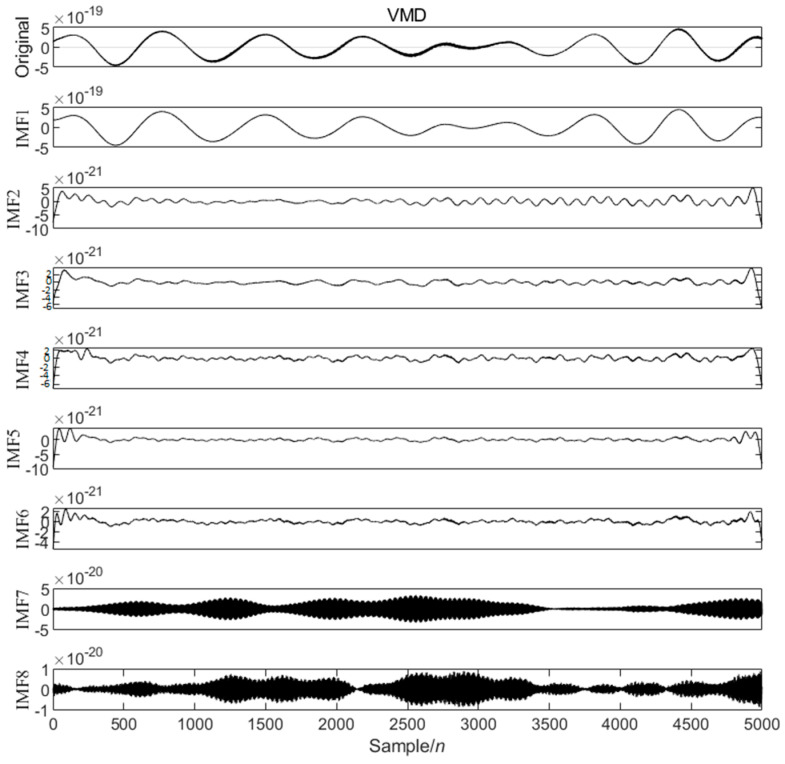
VMD modes of the data segment GW150914 [41001–46000].

Next, based on the characteristics of the second data segment [41001–46000], shown in Figure 11, the PO obtained the optimal number of variational modal decompositions as 8, and the value of α is 1266.0714, decomposing the GW signal into eight modal components, as shown in Figure 12.

**Figure 13:**
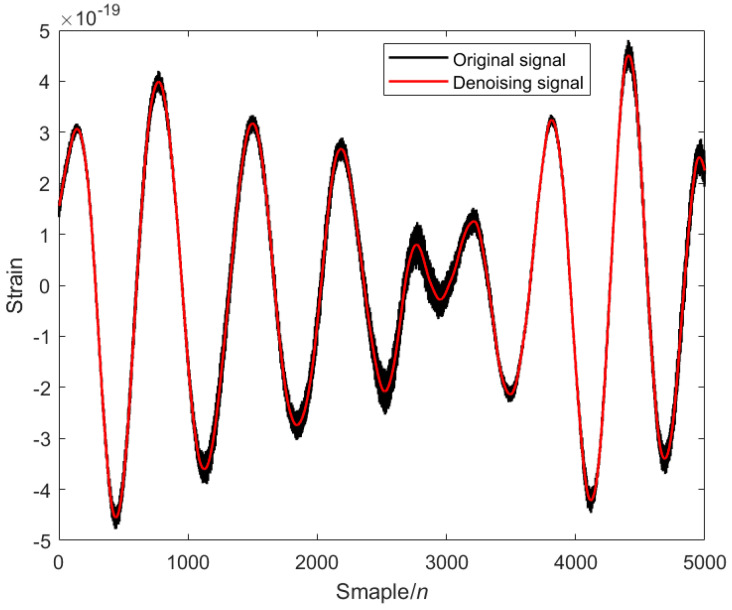
GW150914 [41001–46000] data segment noise-reduction result: black is the original signal, and red is the denoising signal.

From the comparison (Figure 13) of the original signal and the one after noise-reduction processing, it can be seen that the noise in the [41001–46000] GW data segment has been significantly reduced, the quality has been improved, and the waveform characteristics of the GW signal have been successfully retained.

Although the signal processed by PO-VMD can effectively suppress GW noise, the figure shows that there is still residual noise in some parts, especially in the high-frequency part of the signal; compared with other algorithms, IWT performs the weakest in noise suppression. It can be seen from the figure that the noise is still relatively obvious in the signal, and the signal is over-smoothed in some places, causing the loss of detailed information of the GW signal; the PO-VMD-IWT algorithm performs best in recovering GW signals. The noise in the signal is significantly reduced. While reducing noise, the main features and details of the signal are better preserved, the waveform is clear, and the signal is smoother.

**Figure 14:**
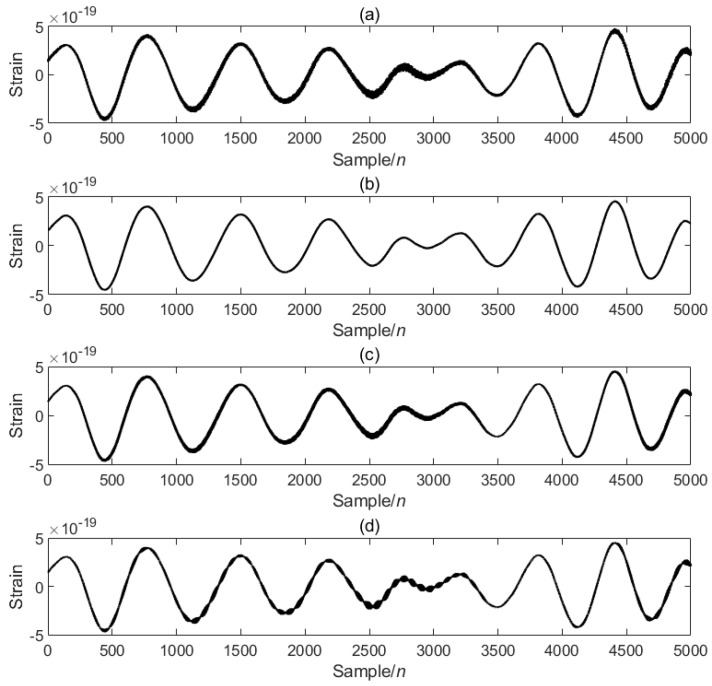
Comparison of various methods for the data segment GW150914 [41001–46000]: (**a**) original signal, (**b**) PO-VMD-IWT, (**c**) PO-VMD, (**d**) IWT.

As can be seen from the comparison in Figure 14 below, the PO-VMD algorithm has a certain suppressive effect on GW noise, but there is still residual noise in some details; IWT has the worst effect. It can be clearly seen that there is a variety of instances of noise interference in the signal, the curve is relatively rough, and some signal details are deformed, affecting the integrity of the signal. Compared with the original signal, the PO-VMD-IWT has significantly suppressed the noise, and the high-frequency noise components mixed in the GW signal are effectively filtered out. The signal waveform is smoother and clearer, and the waveform characteristics of the GW signal are well preserved.

In order to comprehensively evaluate the denoising effects of different algorithms when processing GW signals, we use the widely recognized standard of calculating the signal-to-noise ratio (SNR). Simultaneously, in order to more objectively evaluate the noise-reduction performance of the algorithm, the root mean square error (RMSE) and mean absolute error (MAE) are used. The definitions are as follows:

(17) $$SNR=10\mathrm{lg}\left[\frac{\sum_{n=0}^{N-1}x^{2}(n)}{\sum_{n=0}^{N-1}{\left(x(n)-y(n)\right)}^{2}}\right]$$

(18) $$RMSE=\sqrt{\frac{1}{N}\sum_{n=1}^{N}{\left(x(n)-y(n)\right)}^{2}}$$

(19) $$MAE=\frac{1}{N}\sum_{n=1}^{N}\left|x(n)-y(n)\right|$$

where x(n) is the GW reference signal, y(n) is the GW signal after processed by different algorithms, and N is the signal length.

The Table 1 and Table 2 show the indicators of different algorithms for the GW150914 [22001–27000] and [41001–46000] data segments. It can be seen that the PO-VMD-IWT has the highest signal-to-noise ratio compared with other algorithms, with an average SNR of 38.7273, and the RMSE and MAE are the smallest, both of which are reduced by an order of magnitude, with an average RMSE of 2.67115 × 10^−21^ and an average MAE of 1.8196 × 10^−21^, showing the best noise-reduction performance. PO-VMD has the smallest improvement in SNR and the smallest reduction in mean absolute error. The improved wavelet algorithm improves the signal-to-noise ratio, but the improvement is limited. The data in the table can once again prove the effectiveness of the proposed algorithm.

Experiments show that the use of the PO improves local search accuracy while ensuring global search capability. The VMD optimized by the PO can effectively extract the waveform characteristics of GWs in GW signal processing and significantly suppress background noise interference under low SNR conditions. Combined with the IWT algorithm, the sensitivity and reliability of GW event detection are improved, verifying the superiority of the algorithm in non-stationary signal processing.

## 4. Conclusions

This paper proposes an algorithm for denoising space GW signals based on improved VMD with PO; combined with the IWT, the denoising problem of GW signals is studied in depth. In order to solve the problem of difficulty in separating GW signals from noise, this paper adopts the VMD algorithm to perform modal decomposition processing on GW signals. To solve the problem of difficulty in selecting modal decomposition parameters, the PO is introduced to improve the accuracy of decomposition and the real-time performance of the algorithm, reduce the calculation steps, and improve the processing efficiency. Due to the powerful global search capability of the PO, it helps to avoid falling into the local optima and improve its convergence speed. According to the analysis of experimental data results, compared with other algorithms, the SNR algorithm is significantly improved, and the root mean square error is reduced by one order of magnitude, which shows the effectiveness and superiority of this algorithm and meets the noise-suppression requirements of space GW detection. The proposed algorithm enhances the waveform characteristics of GW signals under background noise, improves the sensitivity and reliability of detection, and provides higher-quality technical support for the research of GW astronomy.

## Figures and Tables

**Table 1:** Comparison of evaluation indicators of various algorithms in the GW150914 [22001–27000] segment.

Algorithm	SNR	RMSE	MAE
IWT	25.3585	1.245 × 10^−20^	9.1358 × 10^−21^
PO-VMD	22.8614	1.6597 × 10^−20^	1.4029 × 10^−20^
PO-VMD-IWT	37.5342	3.0647 × 10^−21^	2.2616 × 10^−21^

**Table 2:** Comparison of evaluation indicators of various algorithms in the GW150914 [41001–46000] segment.

Algorithm	SNR	RMSE	MAE
IWT	25.6452	1.1782 × 10^−20^	8.0893 × 10^−21^
PO-VMD	24.5428	1.3377 × 10^−20^	1.0664 × 10^−20^
PO-VMD-IWT	39.9204	2.2776 × 10^−21^	1.3776 × 10^−21^

## Data Availability

The original contributions presented in this study are included in the article. Further inquiries can be directed to the corresponding author.

## Abbreviations

The following abbreviations are used in this manuscript:

GW	Gravitational Wave
PO	Parrot Algorithm
VMD	Variational Mode Decomposition
IWT	Improved Wavelet Threshold
